# Impact of Sodium on Blood Pressure and Well Being on Coastal Population: A Social Pharmacy Perspective

**DOI:** 10.7759/cureus.103495

**Published:** 2026-02-12

**Authors:** Anisyah Achmad, Thomas Erwin Christian J Huwae, Bagus Putu P Suryana, Faizatul Mukaromah, Intanaya W Nareswara, Sarrah Syifa Azzahra, Umar Idris Ibrahim

**Affiliations:** 1 Department of Pharmacy, Faculty of Medicine, University of Brawijaya, Malang, IDN; 2 Department of Orthopedics and Traumatology, Faculty of Medicine, University of Brawijaya, Malang, IDN; 3 Department of Rheumatology, Faculty of Medicine, University of Brawijaya, Malang, IDN; 4 Department of Pharmacy, Universiti Sultan Zainal Abidin - UniSZA, Terengganu, MYS

**Keywords:** blood pressure, coastal, hypertension, quality of life, salt

## Abstract

Background

Coastal communities often exhibit dietary habits characterized by high sodium intake due to the frequent consumption of salted and preserved foods. Such patterns may contribute to elevated blood pressure and impaired well-being. This study examined the relationship between sodium intake, blood pressure, and health-related quality of life among fishermen living on Madura Island, Indonesia.

Methods

A cross-sectional study was conducted from December 2023 to November 2024 involving 137 adult fishermen who met the inclusion criteria and provided informed consent. Sodium intake was assessed using a coastal-adapted Semi-Quantitative Food Frequency Questionnaire (SQ-FFQ) and quantified using the Indonesian Food Composition Table (TKPI). Blood pressure was measured following standardized procedures using a digital sphygmomanometer. Quality of life was evaluated using the EQ-5D-5L instrument. Spearman correlation tests were used to determine associations between sodium intake, blood pressure, and quality of life.

Results

Most respondents consumed sodium above recommended levels, with 75.18% exhibiting high intake (>1500 mg/day). More than half (54.74%) were categorized as Stage 1 hypertension, and only 32.85% showed normal blood pressure. Quality of life scores indicated mild to moderate limitations, predominantly in the pain/discomfort and usual activities domains. A modest but significant correlation was observed between sodium intake and blood pressure (r = 0.192, p = 0.047), while no meaningful association was found between sodium intake and quality of life (r = -0.015, p = 0.877).

Conclusion

High sodium consumption among Madura fishermen is associated with elevated blood pressure, despite generally preserved quality of life. Routine screening, community-based nutrition education, and targeted interventions promoting reduced-sodium dietary practices are recommended to prevent undiagnosed hypertension and improve long-term health outcomes in coastal populations.

## Introduction

Sodium is an essential mineral crucial for the body because it plays a role in maintaining fluid and electrolyte balance. As the main osmolyte in extracellular fluid, sodium helps regulate body fluid volume and supports muscle function and nerve impulse transmission [1]. Madura Island is known as one of the largest salt-producing regions in Indonesia. According to data from the Ministry of Maritime Affairs and Fisheries in 2021, total salt production in Madura reached over 250 million kilograms, with Bangkalan Regency producing over 6 million kilograms and Sampang Regency over 118 million kilograms [2]. Madura Island's coastal geography means that the majority of its population earns a living as fishermen and salt farmers. Furthermore, the success of the government's salt self-sufficiency program, implemented through intensification, extensification, and pond revitalization strategies, has contributed to the year-over-year increase in salt production in Madura. This high salt production influences people's consumption patterns, particularly in their choice of daily side dishes [3]. Previous research has shown that Madurese people tend to consume seafood such as pindang (spiced fish), tuna (skipjack tuna), and tilapia (mujair), as well as chili sauce made from shrimp paste. These foods are generally salted and therefore contain high levels of sodium [4].

Excessive sodium consumption not only causes electrolyte disturbances and inflammation but is also closely linked to increased blood pressure. Several international studies have confirmed that high sodium intake is a major risk factor for hypertension. The mechanisms underlying the association of high sodium intake with increased blood pressure are associated with water retention, increased systemic peripheral resistance, altered endothelial function, altered structure and function of large elastic arteries, and modified sympathetic activity [5]. When sodium intake is too high, the body automatically retains more water. This causes an increase in blood volume and pressure exerted on the arterial wall and results in constricted blood vessels. According to a 2023 World Health Organization (WHO) report, sodium consumption exceeding 2 grams per day (equivalent to 5 grams of table salt) can increase blood pressure and the risk of cardiovascular diseases such as stroke and heart disease.

The Indonesian Ministry of Health, through Ministerial Regulation No. 28 of 2019, established the Recommended Dietary Intake (RDI), including the maximum daily sodium intake [6]. The standard daily sodium requirement is determined based on age range and gender. The data is presented comprehensively and clearly for public use. To assess sodium consumption patterns, one tool used is the Semi-Quantitative Food Frequency Questionnaire (SQ-FFQ) and then converted to values using the TKPI (Tabel Komposisi Pangan Indonesia) application. Maintaining a balanced daily sodium intake is crucial because both insufficient and excessive intake can lead to various health problems that can reduce a person's quality of life. One instrument for assessing quality of life is the EuroQol 5-Dimension 5-Level (EQ-5D-5L).

Coastal life, which is synonymous with high salt/sodium consumption, needs to be monitored and properly monitored to avoid high incidences of hypertension, which can lead to a decline in the quality of life, both economically and socially. There is an urgency to analyze the relationship between sodium intake with blood pressure and quality of life in the Madurese population [7]. Therefore, this study aims to comprehensively evaluate the relationship between daily sodium intake, blood pressure status, and health-related quality of life among coastal residents of Madura.

The findings of this study are expected to provide evidence-based insights to support targeted public health interventions and nutritional strategies for hypertension prevention and quality of life improvement in coastal communities with high sodium consumption.

## Materials and methods

This study is not a cause-and-effect study, but rather aims to determine the correlation between variables that have a theoretical basis for their interrelationship and can be explained. Therefore, a cross-sectional design was chosen due to time constraints for participants. Furthermore, demographic data collection and questionnaires were sufficient, as they did not require pre- and post-intervention data. The study was conducted at the coastal village hall of Bangkalan, Madura Island, between December 2023 and November 2024. Participants, who were fishermen in Madura Island, were recruited conveniently by the village head. Each fisherman in the area was invited to attend an introductory event for the research project. Fishermen who attended and met the inclusion criteria were given a letter of consent and signed a consent form for participation in the study. Inclusion criteria were: Madurese ethnicity or having resided in Madura for at least one year, at least 25 years of age, male or female, who has not been diagnosed with hypertension, no current medication use, and no nutritional diet. The study was approved by the Research Ethics Committee of the Faculty of Medicine, Universitas Brawijaya, which approved the research protocol (No. 424/EC/KEPK/12/2023).

Data were collected from participants through face-to-face interviews at the research site by enumerators using a questionnaire. In the first part of the questionnaire, participants were asked to provide demographic data, including gender, age, BMI (Body Mass Index), and blood pressure. The second part of the SQ-FFQ is a tool to measure daily sodium consumption (mg) with questions about dietary patterns up to one month prior [8]. This tool is a standard questionnaire for assessing nutritional intake in Indonesia. The SQ-FFQ consists of 33 questions (15 foods, five vegetables, nine drinks, four cooking spices). The selection of food composition in the SQ-FFQ is adjusted to the geographical location of coastal communities (food in coastal environments). To obtain the weighted sodium intake (mg/day), see the TKPI website [9]. The third part of the questionnaire aims to measure quality of life using the EQ-5D-5L questionnaire [10]. The five dimensions of the descriptive section consist of mobility, self-care, daily activities, pain/discomfort, and anxiety. Each dimension is scored on five levels (1-5): no problem, slight problem, moderate problem, severe problem, and extreme problem. Based on self-reported responses, health conditions can be defined as a five-digit number combining the severity of each of the five dimensions, where '11111' represents no problems in any dimension and '55555' represents extreme problems in all dimensions. Blood pressure was measured using a digital sphygmomanometer attached to the left arm, with the respondent seated according to standard operating procedures. Blood pressure data collection was carried out twice, and the largest value was used in calculating the data in this study. All data were collected directly by the enumerator and the researcher during the study.

Descriptive data were calculated as frequencies (%). Prior to analysis, the distributions of sodium intake and systolic blood pressure were evaluated for normality using the Shapiro-Wilk test, and visual inspection of histograms and Q-Q plots revealed mild right-skewness in sodium intake but no extreme outliers; therefore, the Pearson correlation method was applied without transformation. The aim of the study was to determine correlations between variables, so the Spearman test was the appropriate choice, with statistical significance accepted at P < 0.05. Data were analyzed using SPSS version 29.

## Results

A total of 137 participants were recruited from the 160 invited fishermen. Twenty-three fishermen declined to participate due to other commitments, such as family events out of town, work, illness, or not meeting the inclusion criteria.

Most participants were female (76.64%), while males represented the smallest proportion (23.36%). The age distribution showed that the largest group was 56-65 years (42.33%), whereas the youngest group, 26-35 years, accounted for the lowest proportion (2.92%). Regarding nutritional status, the majority of respondents were classified as obesity I (39.42%), followed by Overweight (30.66%), whereas Underweight was the least common category (0.73%). Blood pressure classification revealed that Hypertension Stage 1 was the most prevalent condition (54.74%), while Hypertension Stage 2 accounted for 12.41%. Only 32.85% of respondents had normal blood pressure. Overall, the population was dominated by middle- to older-aged adults, with a high prevalence of overweight and obesity, and a substantial proportion experiencing elevated blood pressure (Table 1).

**Table 1:** Characteristics of respondents

Parameter	Total	
	Subject (n=137)	Percentage (%)
Sex		
Male	32	23.36
Female	105	76.64
Age		
26 - 35 years	4	2.92
36 - 45 years	14	10.22
46 - 55 years	46	33.51
56 - 65 years	58	42.33
≥ 66 years	15	10.95
BMI (Body Mass Index)		
Underweight (< 18.5 kg/m^2^)	1	0.73
Normal (18.5 - 22.9 kg/m^2^)	19	13.86
Overweight (23 - 24,9 kg/m^2^)	42	30.66
Obesity I (25 - 29.9 kg/m^2^)	54	39.42
Obesity II (≥ 30 kg/m^2^)	21	15.33
Blood Pressure (Systolic / Diastolic)		
Normal (< 120-139/ < 80 - 89 mmHg)	45	32.85
Hypertension 1 (140-159/ 90-99 mmHg)	75	54.74
Hypertension 2 (160-179/ 100-109 mmHg)	17	12.41

Based on the TKPI (Indonesian Food Composition Table) guidelines for standard daily sodium consumption (mg/day) by gender and age, the results are shown in Table 2.

**Table 2:** EQ-5D-5L questionnaire results distribution (n=137)

EQ-5D-5L	Level 1 (n)	Level 2 (n)	Level 3 (n)	Level 4 (n)	Level 5 (n)
Mobility	65	39	24	9	0
Self-care	90	40	1	6	0
Usual activities	56	36	41	4	0
Pain/ Discomfort	37	47	43	9	1
Anxiety	92	34	10	1	0

The majority (75.18%, n = 103) consumed sodium at a level categorized as high (>1500 mg/day), with a mean intake of 2,536.88 ± 753.37 mg/day. A smaller fraction (23.36%, n = 32) fell into the low intake category (<1100 mg/day), reporting a mean intake of 326.64 ± 322.78 mg/day, while only two participants (1.46%) were classified in the normalrange (1100-1500 mg/day; mean 1,366.91 ± 139.24 mg/day). The distribution of sodium intake was skewed with large within-group variability, particularly among high-intake participants, indicating a wide range of individual intakes and the presence of high consumers

Assessment of health-related quality of life using the EQ-5D-5L instrument demonstrated varying levels of functional limitation across domains (Table 3). The highest proportion of respondents reported no problems (Level 1) in the anxiety/depression (67.2%) and Self-care (65.7%) domains, indicating generally preserved psychological well-being and independence in basic self-maintenance. In contrast, Pain/Discomfort showed the greatest burden, with more than half of participants reporting Level 2 or Level 3 problems (65.7%), and a small number experiencing severe or extreme discomfort (Level 4 = 6.6%; Level 5 = 0.7%). Usual activities also demonstrated notable limitations, with 29.9% reporting moderate problems (Level 3). Mobility impairments were present in a substantial subset, with 28.5% reporting slight problems (Level 2) and 17.5% moderate problems (Level 3). Overall, while severe and extreme limitations (Levels 4-5) were uncommon across most dimensions, the data indicate considerable mild-to-moderate functional impairment, particularly in the domains of pain/discomfort and usual activities.

**Table 3:** Sodium intake of respondents

Natrium Intake (mg/day)	Subject (n=137)	Percentage (%)	Mean±SD
Low (<1100)	32	23.36	326.64±322.78
Normal (1100-1500)	2	1.46	1366.91±139.24
High (>1500)	103	75.18	2536.88±753.37

Prior to analysis, the distributions of sodium intake and systolic blood pressure were evaluated for normality using the Shapiro-Wilk test, and visual inspection of histograms and Q-Q plots revealed mild right-skewness in sodium intake but no extreme outliers; therefore, the Pearson correlation method was applied without transformation. Results showed a small but statistically significant positive correlation between sodium intake and blood pressure (r = 0.192, p = 0.047), indicating that higher sodium consumption was modestly associated with increased blood pressure, although the effect size was limited. In contrast, sodium intake demonstrated no meaningful relationship with overall quality of life (r = -0.015, p = 0.877).

## Discussion

In this sample of coastal population, adult to elderly individuals, the sodium intake of the majority of participants exceeded the established dietary recommendations. Approximately 75.18% of the respondents consumed an average daily intake of 2536.88 mg of sodium, which is nearly twice the recommended limit by the Dietary Reference Intake for Indonesia [5]. The number of individuals exceeding the recommended salt consumption levels is particularly concerning, given the alarming prevalence rate of hypertension (HT), especially in low- and middle-income countries such as Indonesia [11]. Moreover, the prevalence of HT in this study is approximately 43.92%, which is much higher than Indonesia's prevalence of 30.8% based on the Indonesia National Health Survey [12].

Spearman test analyses revealed the association between sodium intake and blood pressure. Our results suggest that higher sodium intake will increase blood pressure as well. Among dietary components, sodium has been identified as a major contributor to elevated blood pressure levels, particularly when living directly in coastal areas where sodium intake is generally higher [13,14]. These findings are consistent with existing literature, which relates coastal living with increased dietary salt intake from salty foods, a well-documented hypertension risk factor [15]. Coastal populations, including those in Madura, are particularly vulnerable due to their dietary patterns that favor high salt consumption, often through traditional salted fish and other preserved foods [16,17]. The increased risk of hypertension associated with sodium intake observed in this study aligns with the pathophysiological mechanisms of increasing blood pressure. Consumption of excess sodium will increase sodium levels in the blood and disrupt the fluid balance, resulting in water retention, thus leading to a condition of high flow in arterial vessels [5]. The association between dietary sodium intake and blood pressure in the present study may provide important clinical implications for the management of secondary prevention of hypertension.

The findings of this study also suggest a negative correlation between sodium intake and quality of life, although the relationship was not statistically significant. This trend implies that individuals with higher sodium consumption tend to experience a lower quality of life. Excessive sodium intake has been associated with increased fluid retention and vascular resistance, which can contribute to elevated blood pressure and overall cardiovascular burden. This is further supported by the observation that blood pressure rises in parallel with higher sodium intake. Hypertension, a chronic elevation in blood pressure, is a well-established risk factor for the progression of various non-communicable diseases, particularly cardiovascular and renal disorders [18]. Over time, uncontrolled hypertension may lead to serious complications such as stroke, heart failure, or chronic kidney disease, all of which can significantly diminish an individual’s functional capacity and psychological well-being. The reduced quality of life in hypertensive patients may be linked to common symptoms such as fatigue, dizziness, vertigo, or shortness of breath, which can interfere with daily functioning and overall life satisfaction [19]. Although sodium intake may play a role, an individual’s quality of life is also affected by multiple factors such as age, gender, and socioeconomic status, thereby reducing the likelihood that sodium intake alone has a significant influence [20].

Limitations

This study has several limitations that should be acknowledged. First, sodium intake was assessed using the SQ-FFQ, which, although standardized for the Indonesian population, relies on self-reported dietary intake and is susceptible to recall bias, underreporting, and estimation error, particularly in populations consuming home-cooked meals or varying portion sizes. This was addressed by estimating the amount of food consumed through the display of images of small, medium, and large portions. Furthermore, the image display provided participants with a recall of the names of the foods in question.

Second, blood pressure was measured at a single point in time, which may not accurately reflect participants' usual blood pressure levels, especially given daily variability and situational factors such as physical activity or stress. To mitigate data bias, data collection was conducted 15 minutes after participants arrived at the study site.

## Conclusions

In this cross-sectional study of a coastal Madurese population, most participants reported sodium intake above recommended levels and a high prevalence of elevated blood pressure. Sodium intake showed a weak but statistically significant association with blood pressure, while no significant relationship was observed with health-related quality of life. These findings are based on unadjusted analyses and single-time-point measurements and should be interpreted cautiously. The results may be specific to the study sample and do not support causal inferences. Future studies should use a longitudinal design and involve a wider coastal population to increase generalizability.

## References

[1] Strazzullo P, Leclercq C (2014). Sodium. Adv Nutr.

[2] Kementerian Kelautan dan Perikanan (KKP (2025). Annual performance report 2024 Ministry of Fisheries, Aquatic and Ocean Resources. https://kkp.go.id/publikasi/akuntabilitas-kinerja/pelaporan-kinerja.html.

[3] Efendy M, Fajar SR, Farid MF (2014). Mapping the development potential of salt fields in the northern coastal area of Pamekasan Regency. J Kelaut.

[4] Dewi P, Diana Y, Purwidiani N (2015). Study of staple food consumption patterns in coastal communities. E-Journal Boga.

[5] Grillo A, Salvi L, Coruzzi P, Salvi P, Parati G (2025). Recommended nutritional adequacy rate for Indonesian people. Nutrients.

[6] Grillo A, Salvi L, Coruzzi P, Salvi P, Parati G (2019). Sodium intake and hypertension. Nutrients.

[7] (2025). Global burden of disease study 2019 (GBD 2019) data resources. https://ghdx.healthdata.org/gbd-2019.

[8] Shahar S, Shahril MR, Abdullah N (2025). Development and relative validity of a semiquantitative food frequency questionnaire to estimate dietary intake among a multi-ethnic population in the Malaysian Cohort Project. Nutrients.

[9] Mills KT, Bundy JD, Kelly TN (2025). Indonesian food composition table (Tabel Komposisi Pangan Indonesia). Circulation.

[10] Herdman M, Gudex C, Lloyd A, Janssen MF, Kind P, Parkin D, Bonsel G, Badia X (2025). Development and preliminary testing of the new five-level version of EQ-5D (EQ-5D-5L). Qual Life Res.

[11] Mills KT, Bundy JD, Kelly TN (2016). Global disparities of hypertension prevalence and control: A systematic analysis of population-based studies from 90 countries. Circulation.

[12] Farapti F, Fatimah AD, Astutik E, Hidajah AC, Rochmah TN (2025). Health development policy agency team for compiling the 2023 health indicators in figures. J Nutr Metab.

[13] Gupta DK, Lewis CE, Varady KA (2023). Effect of dietary sodium on blood pressure: A crossover trial. JAMA.

[14] Rasheed S, Siddique AK, Sharmin T, Hasan AM, Hanifi SM, Iqbal M, Bhuiya A (2016, 11:). Salt intake and health risk in climate change vulnerable coastal bangladesh: What role do beliefs and practices play?. PLoS One.

[15] Farapti F, Fatimah AD, Astutik E, Hidajah AC, Rochmah TN (2020). Awareness of salt intake among community-dwelling elderly at coastal area: The role of public health access program. J Nutr Metab.

[16] Purwangka F, Mubarok HA (2018). The composition of fish caught using cantrang in the Madura Strait. Albacore.

[17] Utami S, Prasetyo B, Wittiarika ID (2025). The relationship between salted fish consumption habits and the incidence of hypertension during pregnancy in the coastal areas of tuban regency. PHJ.

[18] Kitaoka M, Mitoma J, Asakura H (2016). Erratum to: The relationship between hypertension and health-related quality of life: adjusted by chronic pain, chronic diseases, and life habits in the general middle-aged population in Japan. Environ Health Prev Med.

[19] Snarska K, Chorąży M, Szczepański M, Wojewódzka-Żelezniakowicz M, Ładny JR (2020). Quality of life of patients with arterial hypertension. Medicina (Kaunas).

[20] Kandasamy G, Almanasef M, Orayj K, Alshahrani AM, Alahmari SM (2025). Assessing the impact of hypertension on health-related quality of life: Insights from sociodemographic, economic, and clinical features using SF-36. Healthcare (Basel).

